# Evolutionary analyses of genes in Echinodermata offer insights towards the origin of metazoan phyla

**DOI:** 10.1016/j.ygeno.2022.110431

**Published:** 2022-07-12

**Authors:** Saoirse Foley, Anna Vlasova, Marina Marcet-Houben, Toni Gabaldón, Veronica F. Hinman

**Affiliations:** a Department of Biological Sciences, Carnegie Mellon University, 5000 Forbes Ave, Pittsburgh, PA 15213, USA; b Echinobase #6-46, Mellon Institute, 4400 Fifth Ave, Pittsburgh, PA 15213, USA; c Barcelona Supercomputing Centre (BSC-CNS), Jordi Girona, 29, 08034 Barcelona, Spain; d Institute for Research in Biomedicine (IRB Barcelona), The Barcelona Institute of Science and Technology, Baldiri Reixac, 10, 08028 Barcelona, Spain; e Catalan Institution for Research and Advanced Studies (ICREA), Barcelona, Spain

**Keywords:** Phylogenetics, Phylogenomics, Phylome, Orthology, Echinodermata, Metazoa

## Abstract

Despite recent studies discussing the evolutionary impacts of gene duplications and losses among metazoans, the genomic basis for the evolution of phyla remains enigmatic. Here, we employ phylogenomic approaches to search for orthologous genes without known functions among echinoderms, and subsequently use them to guide the identification of their homologs across other metazoans. Our final set of 14 genes was obtained via a suite of homology prediction tools, gene expression data, gene ontology, and generating the *Strongylocentrotus purpuratus* phylome. The gene set was subjected to selection pressure analyses, which indicated that they are highly conserved and under negative selection. Their presence across broad taxonomic depths suggests that genes required to form a phylum are ancestral to that phylum. Therefore, rather than de novo gene genesis, we posit that evolutionary forces such as selection on existing genomic elements over large timescales may drive divergence and contribute to the emergence of phyla.

## 1. Introduction

The phylum Echinodermata contains several morphologically distinct classes of marine organisms, including sea urchins (Echinoidea), sea cucumbers (Holothuroidea), sea lilies (Crinoidea), and starfish (Asteroidea). As deuterostomes with transparent bilateral embryos, echinoderm systems have been used as models to interrogate developmental questions from the evolution of gene regulatory networks (e.g. skeletogenic processes in [52]; cell type evolution [64], T-box transcription factor binding in [10]) to whole-body regeneration [11,38]. Unlike vertebrates [12], no major genome duplications are known to have occurred within Echinodermata. With an estimated age of over 500 million years [13], this ancient and diverse phylum has been afforded a high level of genomic resource support via Echinobase [6], making Echinodermata an excellent candidate to explore the genomic origins of metazoan phyla.

Recent research has discussed gene loss events as sources of genetic diversity, and thus adaptive evolution (e.g. [1,50]). In particular, a phylogenomic study by Fernández and Gabaldón [18] highlighted the significance of gene loss events in the evolution of metazoan genomes, which was particularly apparent among deuterostomes. However, the effects of deeply taxonomically conserved gene repertoires, particularly those whose functions have not yet been elucidated, have not been investigated by phylogenomic studies thus far. Such genes could prove important in our understanding of both the emergence of phyla and the evolution of function.

In this study, we search for the presence of taxonomically conserved homologous genes of (presently) unknown function across metazoans. We start by deriving a set of such genes across Echinodermata to test whether novel gene synapomorphies contributed to the emergence of the phylum, using a suite of homology prediction tools, gene expression data, and gene ontology. We then generate and report on the phylome of the purple sea urchin (*Strongylocentrotus purpuratus*), which we use in tandem with the aforementioned analyses to inform a set of deeply taxonomically conserved genes of unknown function across metazoans. This gene set is then subjected to selection pressure analyses. We end by commenting on the evolutionary implications of our results, and suggest some related considerations to bear in mind when using homology prediction tools to assess ancient clades.

## 2. Methods

### 2.1. Phylome and species tree reconstruction

The *S. purpuratus* phylome was constructed using the PhylomeDB pipeline [30]. For each gene contained within the *S. purpuratus* genome, a BLAST [5] search was performed against a database containing proteomes of a pan-Metazoan species sampling (Table 1) to retrieve a set of proteins with a significant similarity (e-value < 1e-05, continuous overlap over 50% of the query sequence). The number of hits were limited to the closest 150 hits per gene. A multiple sequence alignment (MSA) was subsequently constructed, where sets of homologous protein sequences were aligned via three different programs: MUSCLE V3.8.1551 [16] (muscle -in File_with_sequences -out Name_outfile), MAFFT v7.407 (mafft –auto File_with_sequences >Name_outfile), and KALIGN v2.04 [34] (kalign -f fasta -i File_with_sequences -out Name_outfile). Both forward and reverse alignments were constructed in each case, and the six resulting alignments were combined using M-Coffee v12.0 [58] (tcoffee File_with_sequences -n_core 1 -output fasta -quiet -aln List_alignment_files -outfile Name_outfile). The resulting alignment was trimmed using trimAl v1.4.rev15 [9] (trimal -compareset paths_to_alignments -forceselect m_coffee_alignment -out intermediate_output -phylip -ct 0.16666 -cons 30; trimal -in intermediate_output -gt 0.1 -cons 30 -out clean_alignment) using a consistency cut-off of 0.1667 and a gap score cutoff of 0.1. The resulting alignments were used to reconstruct phylogenetic trees using IQ-Tree v1.6.9 [41] (iqtree -nt 4 -quiet -mem 4G -cmin 4 -cmax 10 -s clean_alignment -bb 1000 -mset DCmut, JTTDCMut, LG, WAG, VT). The final maximum likelihood (ML) tree was reconstructed using the best model selected based on the Bayesian information criterion (BIC, [49]). Support was calculated using rapid bootstrap (1000 repetitions). Resulting trees and alignments are stored in PhylomeDB 5.0 ([20], http://phylomedb.org), under the phylomeID 349. There are 25,130 trees in total, representing 91.6% of proteome.

Orthology and paralogy relationships were inferred using a speciesoverlap algorithm as implemented in ETE v3 ([31], also see pipeline and commands described in http://etetoolkit.org/docs/latest/tutorial/tutorial_phylogeny.html#species-overlap-so-algorithm). The algorithm traverses the tree and calls speciation or duplication events at internal nodes based on the presence of common species at both daughter partitions defined by the node. This provided the basis to evaluate gene gains and losses. Based on orthology predictions, a gain appears at the common ancestor of each orthologous family and losses appear at nodes where orthologs are missing. Duplication ratios per node were calculated by dividing the number of duplications observed in each node by the total number of gene trees containing that node: the resulting values would either indicate no duplication (value = 0), an average of one duplication per gene in the genome (value = 1), or multiple duplications per gene and node (value >1).

Species trees were reconstructed using two approaches. The first tree was built using DupTree [60] (duptree -i list_trees -o results_duptree.txt 1>duptree.log 2>duptree.error), and was based on all of the genes reconstructed in the phylome. DupTree reconstructs the topology that minimizes the number of duplications when reconciled with a collection of gene trees. The second species tree was built from a selection of 224 genes that had one-to-one orthologs in 14 out of the 15 species; whereby their trimmed alignments were concatenated to yield 122,503 amino acid positions. This alignment was used for approximately-maximum-likelihood tree reconstruction using IQ-Tree [41]. IQ-Tree was run using the same command as per phylome reconstruction, and model selection was performed using IQTREE's ModelFinder, and the LG + F + R7 model was chosen per the BIC criterion.

### 2.2. Mining for taxonomically conserved genes

The dataset generated by Foley et al. [19] was downloaded via Echinobase. This dataset contains one-to-one orthology predictions between *S. purpuratus* and human as called by six different tools: InParanoid v4.1 [44,47] (perl inparanoid.pl proteome1.fasta proteome2. fasta), ProteinOrtho v6 [35] (perl proteinortho6.pl -project=ProjectName -e=1e-40 proteome1.fasta proteome2.fasta), SwiftOrtho [27] (python ./bin/find_hit.py -p blastp -i CatOf2Proteomes.faa -d CatOf2Proteomes.faa -o CatOf2Proteomes.fsa.sc -e 1e-40 -s 111111; followed by python ./bin/find_orth.py -i CatOf2Proteomes.fsa.sc -c 0.5 -y 0 > CatOf2Proteomes.fsa.sc.ort), FastOrtho (http://enews.patricbrc.org/) (./FastOrtho –option_file ../AnalysisDirectory/Analysis_optionfile), OMA ([3], run per DIOPT default), and OrthoFinder v2.4 [17] (./OrthoFinder/orthofinder -f ./DirectoryContainingTwoProteomes). We then extended the searches of InParanoid, OMA, and OrthoFinder to detect orthologs in a variety of other model taxa (*Arabidopsis thaliana, Caenorhabditis elegans, Danio rerio, Drosophila melanogaster, Mus musculus, Rattus norvegicus, Saccharomyces cerevisiae, Schizosaccharomyces pombe*, and *Xenopus tropicalis*), and all tools were run as above. The Foley et al. [19] dataset also contained one-to-one orthology predictions between *S. purpuratus* and members of two other echinoderm classes (Asteroidea and Crinoidea) as called by five of the six tools (all except OMA), which facilitated the subsequent accession of ortholog predictions for both *Acanthaster planci* v1.0 (crown-of-thorns sea star; Asteroidea) and *Anneissia japonica* v1.0 (feather star; Crinoidea). These orthology sets were compared and processed via custom shell scripts to return protein models that were i) not recovered as homologous by any tool in any of the non-echinoderm species, and ii) recovered as orthologous across the three echinoderm species; i.e. the IDs for each of the 1:1 orthologs across our echinoderm dataset were searched for in the non-echinoderm outputs using the "grep" command, and those for which a homolog was detected in this way were excluded from our gene set going forward. The remaining echinoderm-only protein models were entered into the PFAM v33.1 web server [39] for protein domain searching using hmmscan under default conditions [46]. Genes were filtered based on the absence of PFAM gene ontology terms, following which only models that were uncharacterized were retained. Only the longest isoform of each gene was retained.

Per Foley et al. [19], developmental time-course expression data for each of the genes in this remaining subset (i.e. those that matched the homology criterion, lacked PFAM domains, were the longest isoform, and are uncharacterized) was downloaded from Echinobase. Expression data was obtained at the following hours post-fertilization: 0, 10, 18, 24, 40, 40, 48, 56, 64, and 72. We proceeded to further interrogate the activity of genes of interest in *S. purpuratus* by downloading transcriptome data from the SRA-archive corresponding to six different adult tissues corresponding to project PRJNA81157 [56]; gut (SRX173274), ovary (SRX173277), testes (SRX173283), axial gland (SRX173268), radial nerve (SRX173280), and coelomocyte (SRX173270). Transcriptomes were assembled with Trinity V2.11.0 [24] (Trinity –seqType fq –left ../Tissue_1.fastq –right ../data_2.fastq –CPU 20 –max_memory 20G). Protein coding regions were then predicted by Transdecoder [25] (TransDecoder.LongOrfs -t Tissue.trinity.fasta). Each transcriptome was then searched for our genes of interest via BLASTP (1e-7, [8]) (blastp -query GenesOfInterest.fasta -db Tissue.t.fasta.pep -evalue 1e-7 -outfmt 6 > TissueExpression.blast).

The gene expression profiles for the developmental time-course informed our final filtering step. We expect that developmental genes would likely demonstrate specificity within phyla (i.e. derived taxa / clades). Thus, we only retained genes with expression profiles that had a peak transcript per million (TPM) expression value of >50, which comprised the top 10% of our dataset. While there are issues with interpreting expression values from a single dataset [48], we chose this cut-off value in an attempt to remove any genes whose expression signal was potentially comprised of low-level noise. We then subjected the remaining genes to less stringent reciprocal BLASTP searches (1e-7, [8]) to test whether they were synapomorphic for echinodermata. The first BLAST queried these protein models against a set of reference proteomes (as curated by the Quest for Orthologs project) from three other marine organisms; *Nematostella vectensis* v1.0 (starlet sea anemone, Cnidaria), and two early diverging chordates: *Branchiostoma floridae* v1.0 (lancelet, Cephalochordata) and *Ciona intestinalis* HT-version (sea-squirt, Tunicata). The second BLAST queried the entire *Strongylocentrotus purpuratus* proteome against the marine organism proteome set. Commands were per the prior BLAST.

### 2.3. Gene ontology term enrichment

In addition to the PFAM searching during filtering, all *S. purpuratus* genes were annotated using InterProScan v.5.47–82.0 [32] (java -XX:+ UseParallelGC -XX:ParallelGCThreads=4 -Xms128M -Xmx2048M -jar interproscan-5.jar -cpu 4 -d folderName -goterms -pathways -i sequence-file) using all available InterPro databases and scanning applications. Genes associated with the "transposable elements" (TEs) annotation based on the presence of specific PFAM domains were removed from the downstream gene duplication analyses. GO term enrichment analysis between gene lists of interest was run using an in-house Python adaptation of FatiGO [2], and visualized using the REVIGlO [53] server.

### 2.4. Selection pressure assessment

Selected genes of interest that had a corresponding PhylomeDB entry were accessed using PhylomeDB. To ensure a consistent source for subsequent data types, both amino acid and DNA sequences corresponding to these genes were downloaded from Genbank. Amino acid alignments were generated using MAFFT v7.481 [33] (mafft auto—inputorder "input.faa" >"inputAln.faa"). The PAL2NAL v14 package [54] (pal2nal.pl inputAln.faa inputDNA.fas -nogap -output paml > gene. pal2nal) was used to align the DNA sequences codon-by-codon using the amino acid data. Newick trees for each of the genes were accessed via the *S. purpuratus* phylome, and included in the aligned DNA fasta files as a guide for selection analysis in FUBAR [40] via the Datamonkey web application [59]. This enabled the proportions of both positively and negatively selected sites across each gene to be estimated with a posterior probability of 0.95.

## 3. Results

The homology prediction tool suite recovered a set of 145 echinoderm protein models as orthologous across the three echinoderm species, which were not recovered as homologous by any tool in any of the non-echinoderm species. After removing shorter isoforms, and genes with gene ontology annotations, 31 genes remained. Filtering for genes whose TPM values peaked at 50 or more through the developmental time-course yielded a final list of 14 genes of interest that were orthologous among echinoderms, thus far uncharacterized, and expressed throughout development and in adult tissues (full list in supplementary file "GenesAndExpressionData.xlsx"). Reciprocal best hits between *S. purpuratus* and a set of three other marine organisms (*Nematostella vectensis, Branchiostoma floridae, Ciona intestinalis*) were recovered and extracted for 7/14 of these genes (Table 2).

### 3.1. Phylome reconstruction

We reconstructed the evolutionary histories of all genes (phylomes) encoded in the genome of the purple sea urchin (*S. purpuratus*) using the PhylomeDB pipeline [30]. In addition to the purple sea urchin, we selected a pan-phyletic representative set of echinoderm species, together with other species used as an outgroup for phylogenetic tree reconstruction. A complete list of species used in the phylome reconstruction can be found in Table 1. The gene family trees were analysed to predict orthology and paralogy relationships [21], to detect and date duplication events [29], and to transfer functional annotations from one-to-one orthologs (see Methods). All trees and alignments are available through PhylomeDB with the PhylomeID 349 ([20], http://phylomedb.org), which constitutes a valuable resource for researchers interested in the function and evolution of echinoderm genes. Trees are also linked via Echinobase gene pages, and can be accessed in this way.

**Fig. 1.**
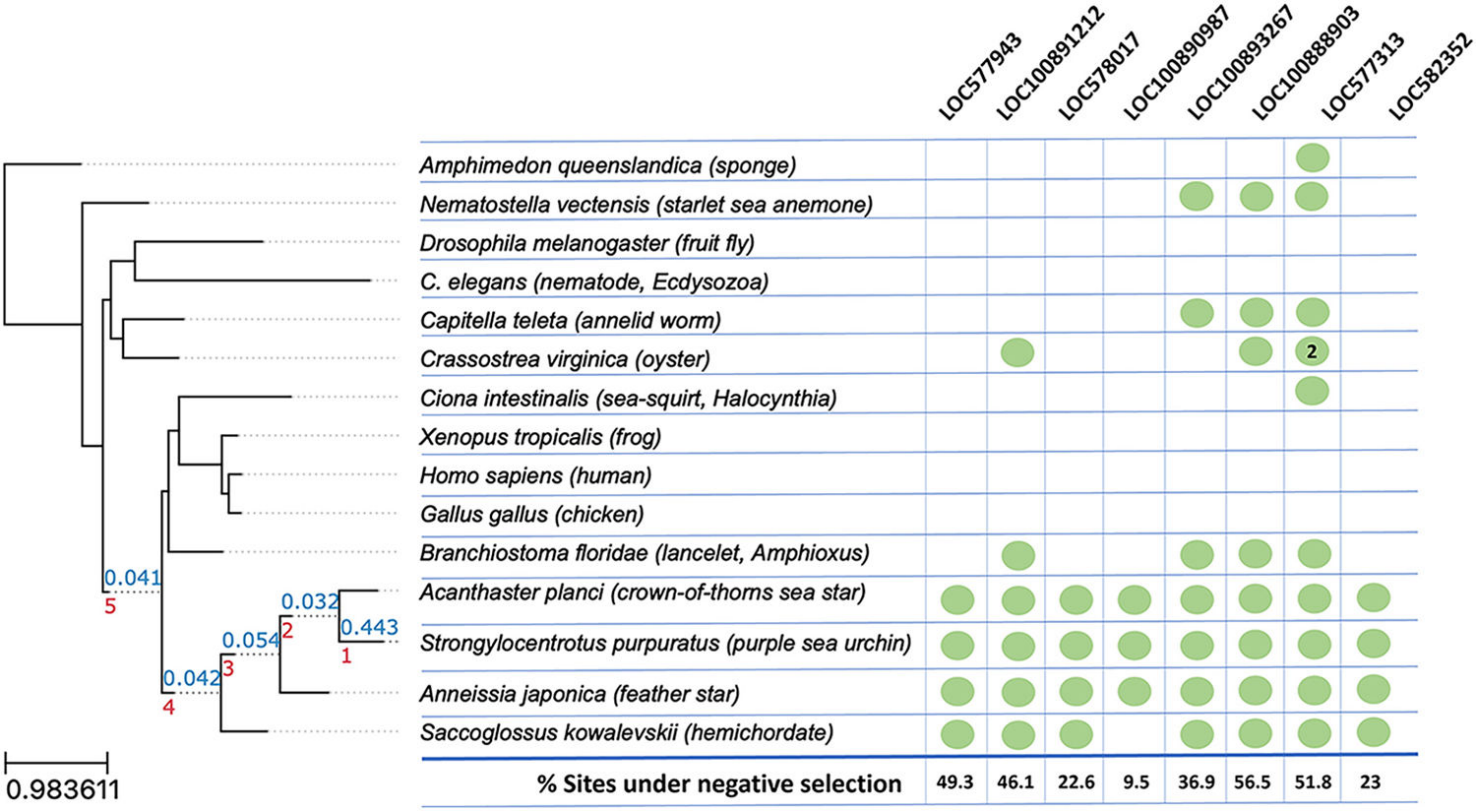
Species tree reconstructed using 224 single-copy genes present in 14 species out of 15 with IQ-Tree software. The red numbers below internal nodes represent the node age. The blue numbers on top represent the duplication rate without large expansions. The tree is appended with each gene of interest for which a PhylomeDB entry was recovered, and the presence of a homolog for that gene is denoted by a green circle. Most of these genes represent single-copy homologs, with the exception of LOC577313, which appears to have been duplicated in the oyster (*Crassostrea virginica*). The percentage of sites under negative selection, as derived using FUBAR, are reported beneath each LOC's column, and demonstrate that most genes are under strong negative selection across a large percentage of its sites. Interestingly, homologs for these genes only appear to be recovered across marine organisms.

**Fig. 2.**
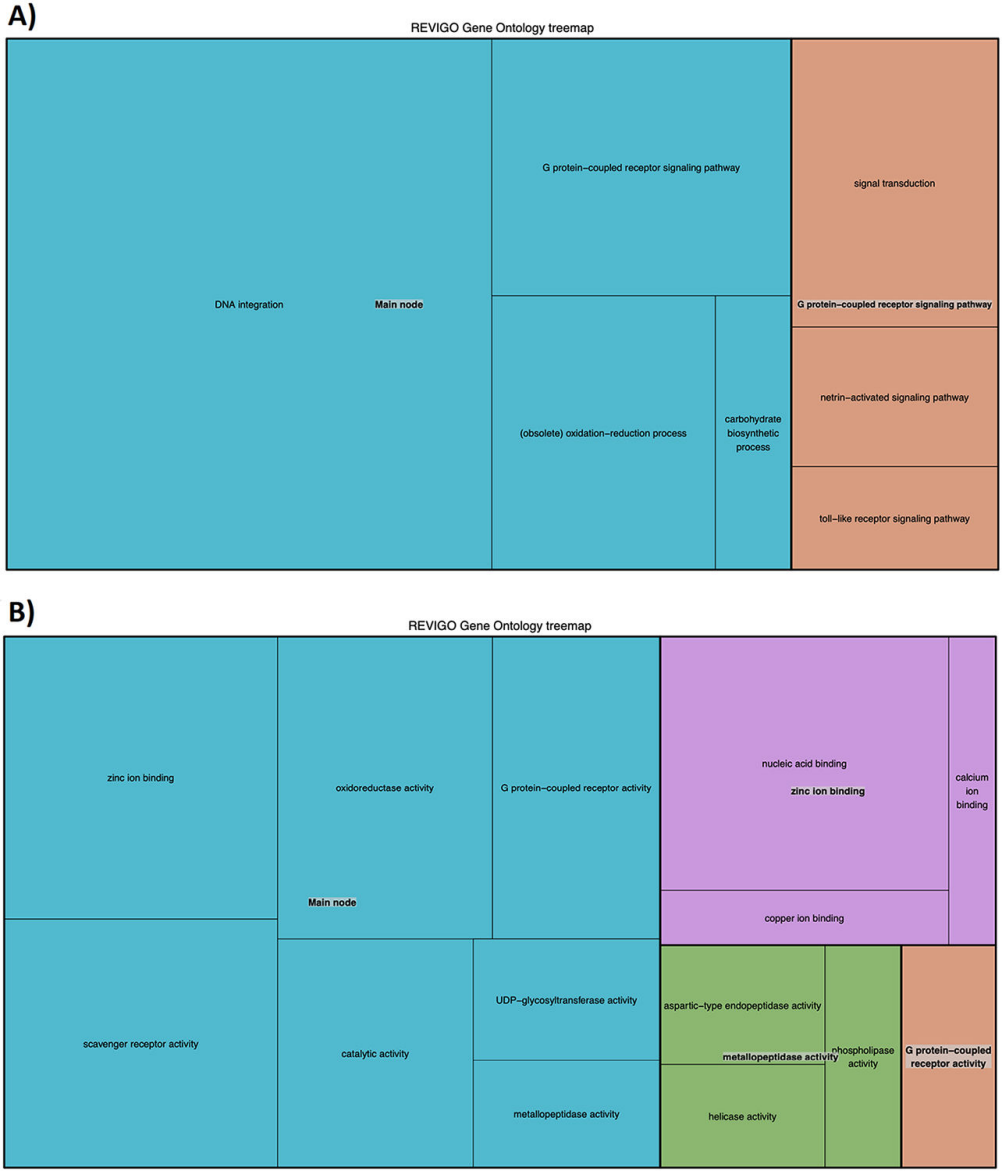
GO term enrichment analysis of the genes duplicated at the *S. purpuratus* level (age #1) for duplications without large expansions, summarized with the REVIGO server. Panel A covers biological processes, and panel B covers molecular function categories.

We reconstructed a species tree representing the evolutionary relationships among the species considered by concatenating the alignments of 224 single copy genes present in at least 14 out of the 15 analysed species (Fig. 1). The resulting phylogeny is fully consistent with the current knowledge of the evolutionary positions of selected species [18]. We used this species tree to compute the duplication densities per branch at each node leading to *S. purpuratus* (Fig. 1) and performed a functional enrichment analysis for genes duplicated at different evolutionary periods (Supplementary file "PhylomeAnalyses.xlsx", depicted in "duplication_by_age.GO_enrichment" tab). The largest gene duplication peak corresponds to the terminal branch specifically leading to sea urchin after its divergence from Asteroidea (represented by the *Acanthaster* lineage), and those genes are enriched in G-protein coupled receptor activity, signal transduction, potassium channel activity, and oxidation-reduction process (Fig. 2).

**Fig. 3.**
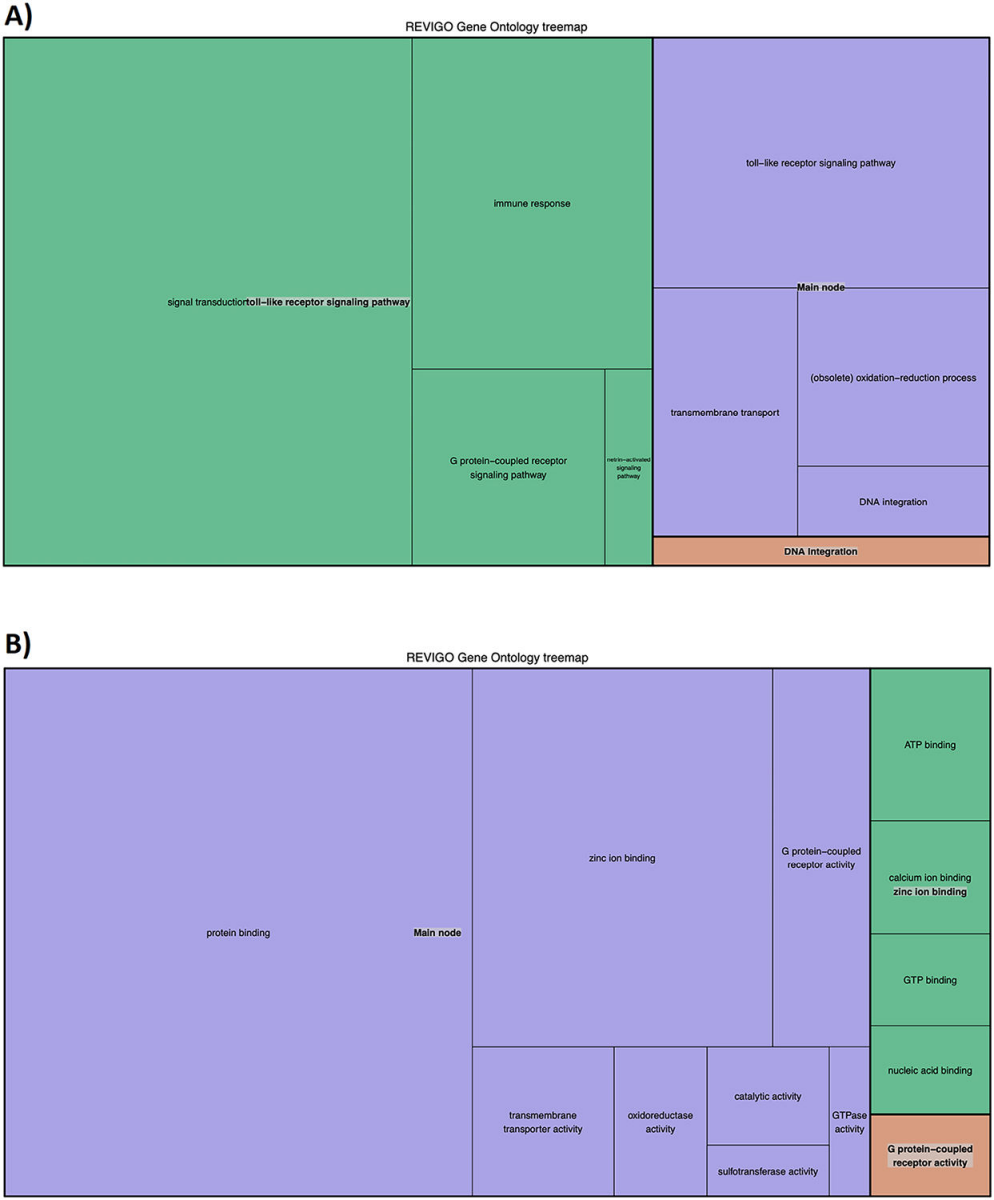
GO term enrichment analysis of the *S. purpuratus* orphan (i.e., potentially lineage specific) genes, summarized with the REVIGO server. Panel A covers biological processes, and panel B covers molecular function categories.

A total of 2758 genes from sea urchin proteome did not have any homologs among selected species (supplementary file "PhylomeAnalyses.xlsx", under the "orphans" tab). Only a small number of these (381) did not have any InterPro signature, including 11 of our 14 genes of interest, indicating they may constitute orphan genes. The remaining were enriched in ontology terms related to signalling receptor activity, immune response, and binding (Fig. 3). Among others, these proteins are enriched in zinc finger domain terms and contain many disordered regions, which is characteristic of transcription factors. Gene trees for 8 of our 14 genes of interest as generated by the PhylomeDB analysis are given in supplementary Figs. S1-S8. No gene trees were recovered for the remaining six genes of interest, as less than 2 homologs were recovered in each case.

We excluded potential transposable elements (TE) based on the presence of specific PFAM domains. In total, we annotated 18,159 proteins as potential TE-related proteins (~4.2%). Nevertheless, we regularly see an enrichment of DNA integration terms in the enrichment analysis.

### 3.2. Spatiotemporal expression

**Fig. 4.**
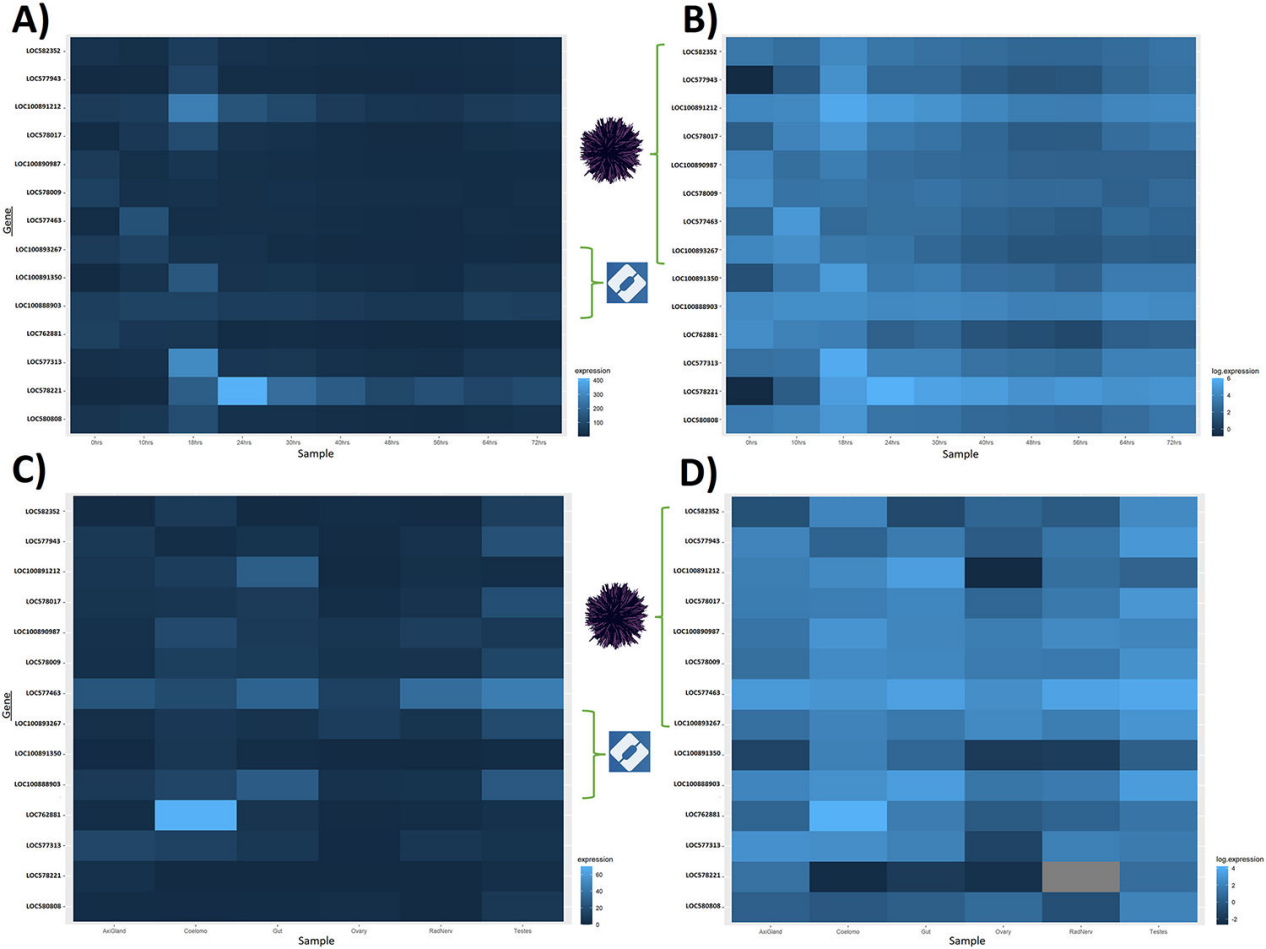
Gene expression in both embryonic and adult tissues represented as a heatmap. In each case, genes in the green bracket corresponding to the purple sea urchin were only recovered in echinoderms, and genes in the green bracket corresponding to the blue InterPro logo were the only ones to record InterPro domains. Panel A shows the raw TPM values for each key developmental timepoint, ranging from 0 to 72 h post-fertilization. Panel B shows the log expression of those TPM values. Expression of our genes of interest is sustained throughout development, generally peaking at 18-24 h. Panel C shows TPM values across six different adult tissues. Panel D shows the log of those values. Expression is also sustained in adult tissues, particularly in the coelomocyte and testes.

Based on the developmental time-course data (supplementary file "GenesAndExpressionData.xlsx"), there were 14 genes that appeared to be expressed and sustained throughout development; 7 that were only recovered in echinoderms, and 7 where BLAST searches matched the hit to another marine organism (Fig. 4). PFAM did not predict any domain hits for these genes, though InterPro predicted domains for one echinoderm-only gene (XM_777691.5 hitting GO:0005515 [glycoprotein binding / protein amino acid binding]), and two marine-organism genes (XM_030995292.1 hitting both GO:0031262 [Nuf2-Ndc80 complex]) and GO:0051315 (attachment of mitotic spindle microtubules to kinetochore; and XM_030999108.1 hitting GO:0005509 [calcium ion binding]).

Gene expression values for the 14 genes of interest in both embryonic and adult tissues are shown in Fig. 4, with raw expression values reported in supplementary file "GenesAndExpressionData.xlsx". These genes are expressed in the embryo throughout development, with most TPM values peaking between 18 and 24 h post-fertilization. Expression of these genes is also detected in all adult tissues, with the testes and coelomocytes (which are the echinoderm immune cells, [51]) showing greater expression values relative to the other tissues.

### 3.3. Selection analysis

Of the eight genes with PhylomeDB entries, each was shown to have a large proportion of sites under strong negative selection across both echinoderm and non-echinoderm taxa (Fig. 1). Raw FUBAR outputs are reported in supplementary Table S1, and report that only a single site in a single gene (LOC577313) is under positive selection. Each of these genes appear to be single copy homologs, with the exception of LOC577313, which is duplicated in the oyster *Crassostrea virginica* (supplementary Fig. S7).

## 4. Discussion

Using genomic data from Echinodermata as a starting point, we identified a set of 14 cryptically homologous genes that are deeply conserved at different taxonomic levels across metazoans, of which 11/14 have no known function as inferred via InterProScan and PFAM ontology enrichment analyses. Given that echinoderms are ancient, first emerging over 500 million years ago [13], and that the genes reported here are also found in non-echinoderm taxa, these genes are ancient, and their origin may pre-date the emergence of Echinodermata—a finding consistent with Tweedt and Erwin [57], who noted that much of the metazoan developmental toolkit was present in early-diverging metazoans before being co-opted for specific developmental functions. This implies that genes necessary to form a phylum are present at the kingdom level, and are thus ancestral to the phylum. Only one gene in our set (LOC578009) had neither a reciprocal best BLAST hit, a gene ontology hit, or a corresponding PhylomeDB tree (i.e. an uncharacterized lineage-specific gene in *S. purpuratus*, Table 2). This gene is worth mentioning, as it may be a candidate for a novel gene synapomorphy in Echinodermata, but it would be the exception to the rule. Our results point towards a lack of novel, synapomorphic genes for Echinodermata. Indeed, this is consistent with a growing body of research suggesting that novel genes may not be the primary drivers of novel features [61].

Despite our gene set being primarily composed of uncharacterized genes of unknown function, the strong negative selection pressure observed across the breadth of various metazoans in the phylome analysis may indicate that these genes are performing important functions at the kingdom level; i.e. perhaps they are under stabilizing selection to ensure that they are retained and do not diversify to any great extent. The expression of these genes in both adult and embryonic *S. purpuratus* tissues further supports their importance. Our results also provide support to an earlier study [18], which showed a general scarcity of gene gain events in most metazoan clades and proposed that gene losses, rather than gene gains, may be the main drivers for clade divergences — a trend that was particularly strong for deuterostomes. Similarly, it is also interesting to note that, per Fig. 1, homologs for our genes of interest only seem to be recovered in marine organisms, which may warrant the assessment of genes loss events in future terrestrialization studies. We therefore suggest that a genomic basis for the emergence of echinoderms and other metazoan phyla may be found not in de novo gene genesis alone, but in the effects of selection pressures exerted upon existing genes and genomic architecture.

While narrowing our list of deeply conserved genes of unknown function, we observed that PhylomeDB and loose reciprocal best BLAST jobs (1e-7) can recover homologs where conventional orthology prediction metrics and tools run under stringent conditions do not. In the case of PhylomeDB, this may be attributed to the composition of the taxon set. The high sequence divergence among our gene set could also be a factor, which may be attributed to a combination of strong positive selection and the large timescale afforded for diversification to occur. This has implications for how we use homology prediction tools when dealing with ancient groups, as several packages rely on sequence similarity and clustering to inform their predictions [23]. The DIOPT [28] approach mitigates this by including several tools that use a combination of clustering and phylogenetic algorithms to make calls on homology, but truly homologous genes may be rendered undetectable when the sensitivity of a conservative analysis is compounded by clade age combined with selection pressures. The performance of different orthology prediction tools in describing the gene content of the last eukaryotic common ancestor has previously been investigated [14], and the associated study noted that (by design) such tools do not return consistent orthogroups when calling distant homologies. That said, emerging methods that are capable of considering protein structure and interactions when calling remote protein homologies seem promising [7,37,62,63], and could prove well suited to predicting distant homologous relationships.

Similarly, it is important to note that novel genes which have evolved via domain shuffling are often missed by orthology prediction tools that search along the entire protein [22]. We likely excluded such genes by prioritizing uncharacterized genes of unknown function (i.e. filtering based on PFAM hits or ontology terms). While tools exist to examine orthology at the domain level (e.g. [45]), there are difficulties in fairly benchmarking them [4]. As such, assessing whether domain shuffling significantly impacts the emergence of phyla may prove fruitful, and genes arising via domain shuffling may prove to be candidates for novel synapomorphic genes among echinoderms. That said, it seems plausible that the effects of positive selection sustained over many millions of years may break down the signatures of domain shuffling via subsequent divergence.

The duplication of transposon genes observed (Fig. 2) may be related to regeneration in echinoderms [15], and we encourage this potential molecular basis for regeneration to be considered in further studies. Given that transposon families are quite diverse, and that DNA integration terms were enriched, this may indicate that TE annotations are underestimated and therefore understudied. Furthermore, it has been shown that genes involved in reproduction and immunity (among others) tend to evolve rapidly in echinoderms [43]. Per our gene expression analysis across adult tissues, our genes of interest are relatively more highly expressed in the testes and coelomocytes, though it is important to note that these values were derived from a single dataset. High expression in the testes is consistent with previous studies [26], and implicates these genes in gamete recognition processes that could promote reproductive isolation [36]. Taken together with the high expression observed in the coelomocytes, which may imply a role in non-host recognition processes, the genes we identify here should be of interest to subsequent studies that seek to understand genomic drivers of speciation.

## 5. Conclusions

By using echinoderms as a starting point and generating the *S. purpuratus* phylome, we recover a set of homologous phylum-level genes of unknown function across metazoans, which may pre-date the emergence of their respective phyla. Novel gene synapomorphies do not appear to have played a role in the emergence of Echinodermata. When investigating distant homologies, our data indicates that more relaxed approaches are better suited to recovering those homologies. We identified strong negative selection across our gene set, which may point towards an attempt to conserve them via stabilizing selection. We subsequently posit that, rather than de novo gene genesis, evolutionary forces on existing genes (such as selection exerted over a large timescale and driving divergence) may have contributed to the emergence of echinoderms and other phyla.

## Supplementary Material

Supplementary Data

GenesAndExpressionData.xlsx

PhylomeAnalyses.xlsx

## Figures and Tables

**Table 1:** List of species used for phylome reconstruction, respectively showing the i) species mnemonic ID, ii) NCBI taxonomy ID, iii) species name, and iv) source for each proteome.

Abbreviation	NCBI taxID	Species	Source
STRPU	7668	*Strongylocentrotus purpuratus*	Echinobase (https://www.echinobase.org/entry/
ACAPL	133434	*Acanthaster planci*	OIST Marine genomics https://marinegenomics.oist.jp/gallery
1,529,436	1529436	*Anneissia japonica*	NCBI, PRJNA615663
CIOIN	7719	*Ciona intestinalis*	Quest For Orthologs 2008
NEMVE	45351	*Nematostella vectensis*	Quest For Orthologs 2020
BRAFL	7739	*Branchiostoma floridae*	OIST Marine genomics https://marinegenomics.oist.jp/gallery
AMPQE	400682	*Amphimedon queenslandica*	Ensembl Metazoa
CRAVI	6565	*Crassostrea virginica*	NCBI, PRJNA376014
283,909	283909	*Capitella teleta*	NCBI, PRJNA175705
CAEEL	6239	*Caenorhabditis elegans*	Quest For Orthologs, 2020
XENTR	8364	*Xenopus tropicalis*	Xenbase (http://www.xenbase.org
CHICK	9031	*Gallus gallus*	Quest For Orthologs, 2020
DROME	7227	*Drosophila melanogaster*	Quest For Orthologs, 2020
SACKO	10224	*Saccoglossus kowalevskii*	OIST Marine genomics https://marinegenomics.oist.jp/gallery
HUMAN	9606	*Homo sapiens*	Quest For Orthologs, 2020

**Table 2:** Our gene set. Ontology is reported by InterProScan. Cases where the phylomeDB ID is followed by an asterisk (*) indicate that the gene has no corresponding phylomeDB tree (e.g. this may happen when fewer than two homologs were recovered). LOC578009 is the only putatively novel synapomorphic gene recovered for echinoderms, but represents an exception; these genes are largely found across metazoans.

Gene	RBBH	PhylomeDB ID	Ontology
LOC100890987	N/A	Phy00E9VCL_STRPU	N/A
LOC100891212	N/A	Phy00E9NDK_STRPU	N/A
LOC577463	N/A	Phy000VU63_STRPU*	GO:0005515
LOC577943	N/A	Phy00E9SJD_STRPU	N/A
*LOC578009*	*N/A*	Phy000VM39_STRPU*	*N/A*
LOC578017	N/A	Phy00E9SMM_STRPU	N/A
LOC582352	N/A	Phy00E9ZEJ_STRPU	N/A
LOC762881	C3YW32_BRAFL	Phy000VNKZ_STRPU*	N/A
LOC100893267_iso_X1	C3YI62_BRAFL	Phy00E9SFI_STRPU	GO:0031262, GO:0051315
LOC100888903	A7S6U8_NEMVE	Phy00EA5U1_STRPU	N/A
LOC100891350	A7SDT1_NEMVE	Phy00EA8QT_STRPU*	GO:0005509
LOC580808	A7S7R1_NEMVE	Phy0037CV2 STRPU*	N/A
LOC577313	F6REZ6_CIOIN	Phy00EAAOU_STRPU	N/A
LOC578221	C3ZA97_BRAFL	Phy000VYP2_STRPU*	N/A
