# Supplementary material for: Evolutionary analyses of genes in Echinodermata offer insights towards the origin of metazoan phyla
Source: Genomics. Author manuscript; Available in PMC 2022 Oct 11. (PMC9552553; doi:10.1016/j.ygeno.2022.110431)
Supplement: Supplementary Data [file NIHMS1838243-supplement-Supplementary_Data.docx]

**Supplementary data for Foley et al.;**

**“Evolutionary analyses of genes in Echinodermata offer insights towards the origin of metazoan phyla”**

Supplementary figures S1-S7 are gene trees from our resulting phylome analysis, accessed from the PhylomeDB database (<http://beta.phylomedb.org>) under the phylomeID 349.

Figure S1: Gene tree for LOC577943


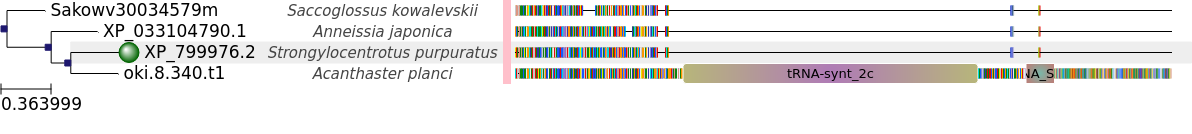


Figure S2: Gene tree for LOC100891212


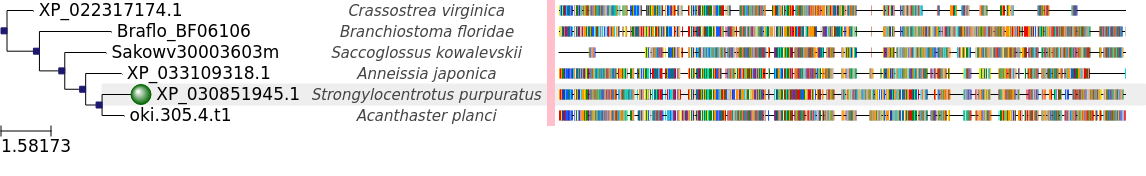


Figure S3: Gene tree for LOC578017


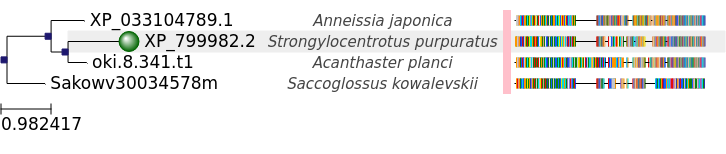


Figure S4: Gene tree for LOC100890987


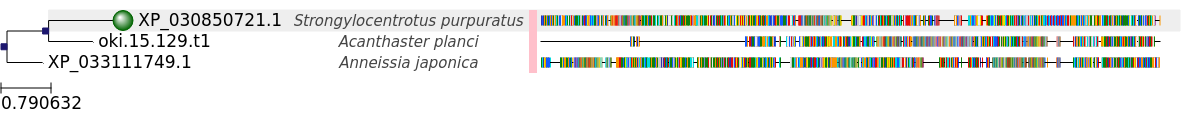


Figure S5: Gene tree for LOC100893267


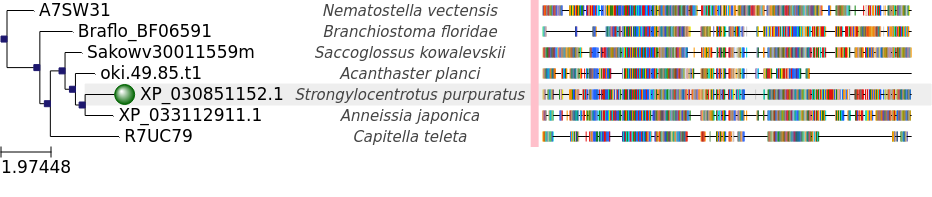


Figure S6: Gene tree for LOC100888903


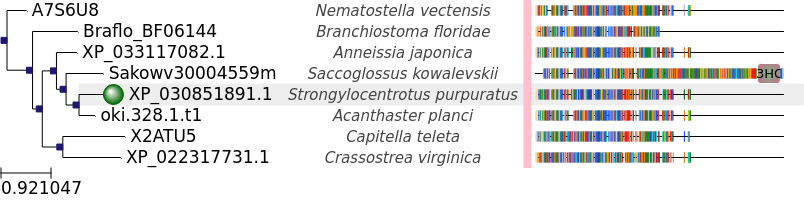


Figure S7: Gene tree for LOC577313


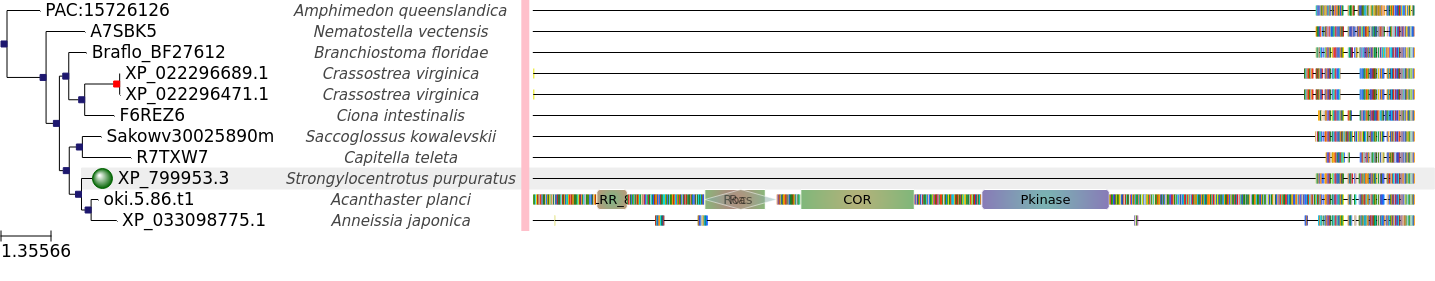


Figure S8: Gene tree for LOC582352


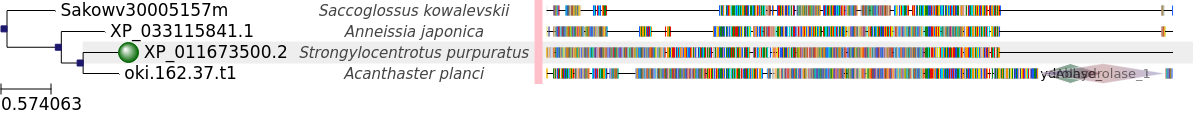


Table S1: FUBAR outputs for each gene of interest, showing the distribution of positively and negatively selected sites with a posterior probability of 0.95.

| **LOC ID** | **TOTAL SITES** | **#POSITIVE** | **#NEGATIVE** | **%NEGATIVE** |
| --- | --- | --- | --- | --- |
| LOC577943 | 290 | 0 | 143 | 49.3% |
| LOC100891212 | 550 | 0 | 254 | 46.1% |
| LOC578017 | 274 | 0 | 62 | 22.6% |
| LOC100890987 | 916 | 0 | 87 | 9.5% |
| LOC100893267 | 268 | 0 | 99 | 36.9% |
| LOC100888903 | 221 | 0 | 125 | 56.5% |
| LOC577313 | 106 | 1 | 55 | 51.8% |
| LOC582352 | 213 | 0 | 49 | 23% |
